# Plant growth, salt removal capacity, and forage nutritive value of the annual euhalophyte *Suaeda salsa* irrigated with saline water

**DOI:** 10.3389/fpls.2022.1040520

**Published:** 2023-01-17

**Authors:** Ning Wang, Zhenyong Zhao, Xinyi Zhang, Sihai Liu, Ke Zhang, Mingfang Hu

**Affiliations:** ^1^ College of Resources and Environment, University of Chinese Academy of Sciences, Beijing, China; ^2^ Xinjiang Institute of Ecology and Geography, Chinese Academy of Sciences, Urumqi, China

**Keywords:** halophyte, *Suaeda salsa*, brine irrigation, revegetation, saline–alkali soil, forage nutritive value

## Abstract

Sustainable agricultural development in semiarid and arid regions is severely restricted by soil and water salinization. Cultivation of the representative halophyte *Suaeda salsa*, which can be irrigated with saline water and cultivated on saline soils, is considered to be a potential solution to the issues of freshwater scarcity, soil salinization, and fodder shortage. However, the salt removal capacity and differences in the forage nutritive value of *S. salsa* under different saline water treatments remain unknown. Using the methods of field trials and randomized blocks design, we quantified salt accumulation in the aboveground biomass, and the biochemical and nutritive value of field-cultivated *S. salsa* in arid northwestern China under irrigation with water of different salinities [i.e., freshwater or water containing10, 20, 30, or 40 g/L NaCl). The fresh and dry weights of *S. salsa* increased, then decreased, with increase in salinity. The salt content of the plant’s aboveground biomass increased to a constant range and, thus, the salt extraction of *S. salsa* was relatively stable under different salinities of irrigation water. Under the experimental conditions, the crude protein content significantly increased to 9.45% dry weight (DW) and then decreased to 6.85% DW, with an increase in salinity (*p* < 0.05). The neutral detergent fiber (42.93%–50.00% DW) and acid detergent fiber (34.76%–39.70% DW) contents were suitable for forage. The contents of trace elements, such as copper and zinc, were significantly increased after irrigation with saline water (*p* < 0.05). The forage of *S. salsa* is of high nutritive value for livestock, and contains low concentrations of anti-nutrients. Therefore, *S. salsa* can be considered for cultivation in saline soils irrigated with saline water. In addition, it provides a viable additional source of fodder in arid regions, where the availability of freshwater and non-saline arable land is limited.

## 1 Introduction

Global livestock production has rapidly expanded in recent years, with much of the increase occurring in China. In this context, the demand for forage grain is increasing (Zhou et al., 2008). The rising global food demand will be difficult to meet with existing agricultural systems because an equivalent increase in agricultural land area is not possible (Al-Azzawi and Flowers, 2022). Water scarcity and salt stress are crucial factors that contribute to the scarcity of pasture resources in arid and semiarid areas of the world (Agudelo et al., 2021). Reported potential yield losses are estimated to be 17% and 20% under drought and salinity, respectively (Hedayati-Firoozabadi et al., 2020). In arid and semiarid climates, including low-rainfall saline regions of Australia, the USA, many Asian countries, and the Mediterranean region, the combination of high levels of water evaporation, low rainfall, irrigation with low-quality water, and other irrational anthropogenic activities results in soil salinization and good-quality forage for livestock being in short supply (Hessini et al., 2020). However, these areas are important for agricultural development and the ecological environment, especially in developing countries. For example, many of the saline–alkali areas in China have a small human population and underdeveloped industrialization, thus making them highly suitable for large-scale animal husbandry (Liu and Wang, 2021). However, conventional forage grows slowly or cannot survive in saline–alkali soil. Therefore, the integration of salt-affected soils and saline water resources to enable sustainable agricultural production with constrained resources is an important problem requiring urgent resolution.

Halophytes, which constitute 1% of the global flora (Flowers and Colmer, 2008), can produce relatively high quantities of consumable biomass in saline areas where non-halophytic species cannot grow or produce only low dry-matter yields (El Shaer, 2010). In addition, in general, the forage produced from saline land has higher forage nutritive values and can improve the quality, nutritional composition, and weight of meat from cattle and sheep (Sun et al., 2015; Ali et al., 2016). Hence, the use of naturally salt-tolerant species to provide forage resources in arid and saline environments is an emerging agricultural strategy (Hessini et al., 2020). This approach has been applied worldwide. For example, Australia is classified as the world’s driest continent and is challenged by soil salinization (Ben Salem and Morand-Fehr, 2010). In this context, various halophytes are now widely accepted as forage plants. There are some relatively successful examples of the integration of halophytes and salt-tolerant forage into small-ruminant production systems (Norman et al., 2010). In low-rainfall saline regions of the USA, Mexico, and many Asian countries, water for agricultural use is extremely limited. Therefore, local agriculture largely depends on the use of marginal water sources, such as saline water and seawater, and drives the cultivation of plants that can tolerate salt water for a variety of uses (Ali et al., 2016; Ozturk et al., 2018; Garza-Torres et al., 2020; Hedayati-Firoozabadi et al., 2020). Salinity and aridity are also encountered in Mediterranean countries. There are many halophytes that grow naturally in almost all estuarine, lagoon, and coastal ecosystems; some of these species have been cultivated as alternative vegetable crops and forage for animal consumption (e.g., 20% and 9% of Iberian halophytes, respectively) (Duarte and Cacador, 2021).

However, achieving grazing value from saline systems is not straightforward (Norman et al., 2013). Different species respond to salinity in different ways because salt tolerance varies greatly among halophytes (Fita et al., 2015; Kumari et al., 2015; Himabindu et al., 2016). It is reported that chenopods are generally more salt tolerant than other halophytic grasses and legumes, and have high crude protein and mineral contents, which are important for ruminant production (Norman et al., 2013). Therefore, chenopods are suitable for use as a forage reserve during drought, or as a supplementary feed source in arid and semiarid environments.

*Suaeda salsa*, an annual euhalophytic herb, and a member of the Chenopodiaceae family, is widely distributed in the intertidal zone and inland saline sites in China (Zhao et al., 2018; Wang and Song, 2019). The succulence and salt absorption capacities of halophytic plants allow them to thrive in high-salinity habitats and to be pioneer plants in saline–alkali lands (Wu et al., 2012; Xu et al., 2013; Song and Wang, 2015). Hence, *S. salsa* is considered to be a promising model plant to enable us to understand salt tolerance and to develop saline agriculture with halophytes (Song and Wang, 2015). *Suaeda salsa* can provide high-quality forage (Li et al., 2017). Wei (2016) reported that the inclusion of an appropriate proportion of *S. salsa* forage can improve pig fattening performance, provide economic benefits, and help to improve immunity. Zhang et al. (2018) reported that *S. salsa* can increase the daily gain of Altay sheep, reduce the feed conversion ratio, increase the net meat yield, and reduce the carcass fat percentage. In addition, *S. salsa* is of high ecological value and is widely suitable as a pioneer species for saline–alkali vegetation restoration. Harvesting of the aboveground biomass of *S. salsa* can significantly decrease the salt content of saline soils. *S. salsa* has also been proposed to be a biomaterial useful in the removal of heavy metals from heavy metal-contaminated soils, especially contaminated saline soils (Wang and Song, 2019; Shang et al., 2020).

Given these attributes, we consider that *S. salsa* may be suitable as a halophytic forage and as an aid to the process of revegetation. However, forage mass and survival alone do not adequately describe its grazing potential on saline rangelands (Waldron et al., 2020). Environmental factors affect plant growth rate and forage nutritive value and production by altering the physiological processes of plants (Etesami and Beattie, 2018). Considering the unique physiological characteristics of halophytes, the effect of salt ions and certain secondary compounds on forage nutritive values should also be taken into account (Masters et al., 2007; Munns and Tester, 2008). Under severe salt stress, halophytes can absorb and synthesize ions and solutes to maintain osmotic pressure, and protein and fiber synthesis may be affected (Muchate et al., 2016). In addition, antioxidants synthesized by halophytes to detoxify reactive oxygen species are critical to forage nutritive values (Norman et al., 2013; Wang et al., 2020). Therefore, whether or not *S. salsa* can be used as forage and how its forage nutritive value changes under irrigation with high-salinity water are unclear. Further evaluation of the effect of salinity on its forage nutritive value is needed.

Hence, a field study was conducted to determine the yield, forage nutritive value indicators [including the contents of ash, crude protein, crude fat, minerals, neutral detergent fiber (NDF), and acid detergent fiber (ADF)], and selected secondary metabolites of *S. salsa*. This research aimed to address the following questions: (i) From an economic perspective, under irrigation with highly saline water, can *S. salsa* survive and produce adequate biomass, and how is its forage nutritive value affected? (ii) With regard to the rehabilitation and improvement of natural ecosystems, does the salt removal capacity of *S. salsa* vary with different degrees of salinity? The results will enrich knowledge of the utilization of saline land and water resources, and provide a scientific basis to promote the development of local animal husbandry in saline environments.

## 2 Materials and methods

### 2.1 Study location

The study was conducted in saline wasteland in the Agricultural Comprehensive Development Zone of Karamay, Xinjiang, China, from June to November 2021. The development zone is located on the lacustrine plain 10 km southeast of Karamay, and has a temperate desert climate. In the summer, this area is extremely hot, with a maximum average temperature of 49.1°C, and in winter it is cold, with a minimum average temperature of –42.0°C (Qiao et al., 2011). The average annual precipitation is 108.9 mm, and the average annual evaporation is 3,008.9 mm. Irrigation water in the experimental area was obtained from a reservoir in the western suburb of Karamay; the water quality properties are summarized in **Table 1**. The physicochemical attributes of the soil in the experimental field before the study are listed in **Table 2**.

**Table 1:** Chemical characteristics of irrigation water (g/L).

pH	K^+^	Na^+^	Ca^2+^	Mg^2+^	Cl^–^	SO_4_^2–^	HCO_3_^–^	CO_3_^2–^
7.07	0.004	0.020	0.034	0.006	0.022	0.070	0.083	0.000

**Table 2:** Initial salt content and ion composition of soil.

Depth (cm)	Salinity (g/kg)	Moisture (%)	Na^+^ (g/kg)	K^+^ (g/kg)	Ca^2+^ (g/kg)	Mg^2+^ (g/kg)	Cl^–^(g/kg)	SO_4_^2–^(g/kg)	HCO_3_^–^(g/kg)
0–20	3.62	13.00	0.68	0.03	0.28	0.17	0.59	1.56	0.27
20–40	8.77	19.14	1.89	0.04	0.69	0.28	2.48	2.55	0.23
40–60	7.46	21.38	1.49	0.04	0.72	0.21	2.18	2.15	0.24
60–80	5.01	23.63	1.07	0.03	0.35	0.15	1.17	1.69	0.26
80–100	4.96	23.12	0.69	0.03	0.59	0.18	1.30	1.45	0.24

### 2.2 Plant irrigation and soil salinity

**Figure 1:**
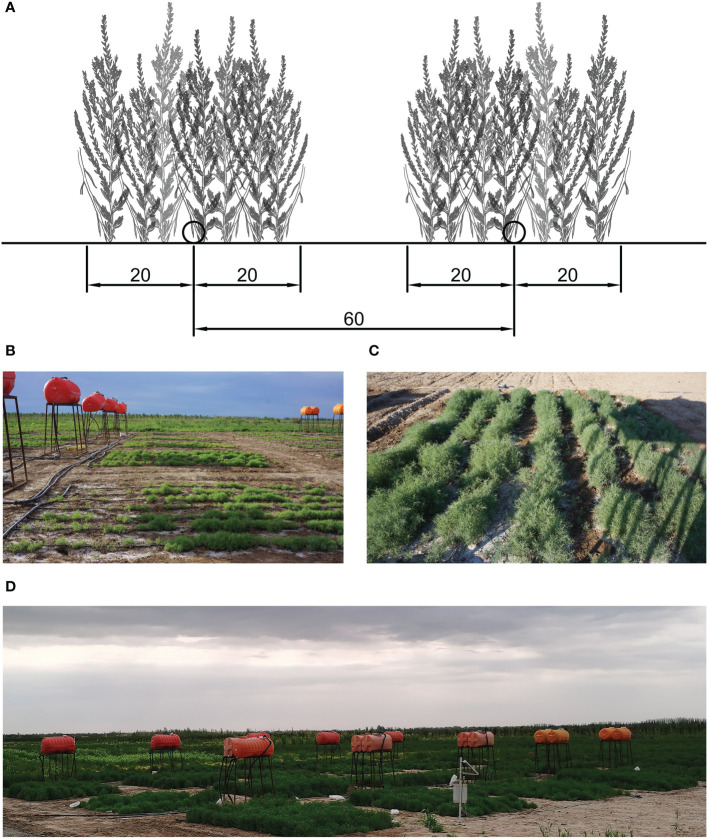
**(A)** Planting design used in the study. The distance between adjacent capillary belts was 60 cm and the planting belt was 40 cm in length. **(B)** Irrigation system consisting of water tanks, polyvinyl chloride pipes, ball valves, water meters, and capillaries. **(C)** View of one experimental plot. **(D)** View of the overall experimental area.

The experiment consisted of five treatments according to different saline contents of irrigation water, consisting of CK and water containing 10, 20, 30, and 40 g/L NaCl. All treatments were replicated four times, and 20 experimental plots were arranged in a randomized block design to reduce the influence of one-way soil difference on the experiment (**Figure 1D**). Freshwater was drawn from the local drip irrigation system and mixed with different amounts of NaCl to achieve the required salinity. Each treatment was equipped with an independent gravity drip irrigation system, consisting of a water tank (2 m above the ground), polyvinyl chloride pipes, ball valves, water meters, and capillaries (**Figure 1B**).

Each plot was 5 m long and 6 m wide (**Figure 1C**). The distance between adjacent capillary belts was 60 cm. The *S. salsa* seeds, fine sand, and water were mixed into loose grains and sown at a depth of 20 cm on both sides of the capillary to form a 40-cm seeding belt (**Figure 1A**). The seeding belts were irrigated immediately after sowing. Thereafter, irrigation was carried out every 2 d. At the seedling stage, irrigation was carried out based on the soil condition. Irrigation was carried out until overlap was attained in the humidification ranges between adjacent capillary belts. All plots were irrigated using the same irrigation interval and irrigation volumes.

### 2.3 Plant material, sampling, and growth measurements

A 0.6 m × 0.6 m quadrat was randomly selected in each plot, reflecting the distance between adjacent capillary belts. A previous report indicates that the maximum forage nutritive value of *S. salsa* is reached during its initial flowering stage (Li et al., 2017). Therefore, sampling determined for forage nutritive value was performed at this stage. The fresh weight (FW) was determined using a balance. The plant was then placed in an oven for fixation at 105 ± 2°C for 30 min and dried at 60 ± 2°C until constant weight, and weighed again to determine the dry weight (DW). The yield per hectare, including FW and DW, was estimated in proportion to the sampled area. The fixation process can rapidly inactivate biologically active enzymes by way of thermochemical reactions. Thus, fixation is widely used as the primary processing method for using plants. Fixation before dying can reduce the loss of active component content. The plant height (cm) was measured from the soil surface to the highest shoot tip. Succulence was calculated as the FW to DW ratio.

The ash content was measured based on Chinese national standard GB/T 64382007. The crude protein (CP) content was determined based on national standard GB/T 6432-2018 using the Kjeldahl method. The van Soest method was used to determine the NDF and ADF content (Van Soest, 1963). In accordance with national standard GB 5009.268-2016, the contents of K, Ca, Na, Mg, and P were determined by inductively coupled plasma emission spectrometry (ICP-OES). The contents of Fe, Zn, Cu, Mo, Ni, Cr, Cd, As, Pb, and Hg were determined by inductively coupled plasma mass spectrometry (ICP-MS). The contents of Cl and oxalate were determined by ion chromatography using the following specific equipment and conditions: AS-LH-AC-3 chromatographic column (250mm * 4.6 mm); column temperature 35°C; mobile phase 2 mmol/L Na_2_CO_3_ and 10 mmol/L NaHCO_3_; and flow rate 1.5 mL/min.

The content of S was determined using an elemental analyzer (Vario MICRO Cube, Germany). We followed the operating conditions and instructions recommended by the manufacturer, which are as follows: oxidation tube temperature 1,150°C; reduction tube temperature 850°C; helium pressure 1,200 mbar; helium flow rate 230 mL/min; oxygen flow rate 45 mL/min; the absorption and release temperature of the CO_2_ absorption column were 40°C and 60°C, respectively; the absorption and release temperature of the water absorption column were 40°C and 140°C, respectively; and the absorption and release temperature of the SO_2_ absorption column were 40°C and 240°C, respectively.

The betaine concentration was determined using ion chromatography in accordance with national standard GB/T 23710-2009. The tannin content was determined using UV–Vis spectrophotometry (8453, Agilent). Samples of about 2–5 g were weighed, then washed in a 100-mL volumetric flask with 80 mL of water. We then placed the extract in a boiling water bath for 30 min, and completed to 100 mL measuring flask by deionized water. A 2-mL sample extract was absorbed, then centrifuged at 8,000 rev/min for 5 min at room temperature. The supernatant was removed and stored. The following was added to 1 mL of clear supernatant: 5.0 mL of water, 1.0 mL of a sodium tungstate–sodium molybdate mixed solution, and 3.0 mL of a sodium carbonate solution. After being set aside for 2 hours, the absorbance of the sample solution was determined at 765 nm, with the standard 0.00 mg/L used as a blank. The tannin concentration of the sample solution was calculated and expressed as mg/L gallic acid equivalent from the standard curve.

Soil column samples of 0–40 cm were taken with a soil drill. We allowed the air-dried soil samples to pass through screens with an opening size of 2 mm. We then weighed a 5.0-g soil sample, put it in a 250-mL flask and added 50 mL of NH_4_OAc solution. After shaking for 30 min, samples were filtered through dry qualitative filter paper. The Na concentration in the filtrate was determined using a flame photometer.

The ash accumulation was calculated as the product of ash content and aboveground biomass. *Suaeda salsa* can absorb salt from the rhizosphere and salt also accumulates in its aboveground plant tissues. Thus, this portion of the salt is removed from the soil when the aboveground biomass is harvested, and the ash accumulation represents its salt removal capacity.

In accordance with Antisari et al. (2020), the salt bioaccumulation factor (BCF) was calculated as the ratio of the Na content of aboveground biomass to the Na concentration in the rhizosphere.

### 2.4 Statistical analysis

Figures were prepared using Microsoft Excel 2016. Statistical analyses were conducted using SPSS 24.0 software. All data were analyzed by one-way analysis of variance (ANOVA), with the salinity of irrigation water as the independent variable. The response variables for these ANOVAs were growth, composition, ion content, and secondary metabolites concentration. Tukey’s honestly significant difference test was used for a comparison of means. The significance was determined at the 5% significance level. All results are presented as means plus or minus the standard error (SE). Histograms were drawn using OriginPro 2021 software.

## 3 Results

### 3.1 Growth of *S. salsa*

**Figure 2:**
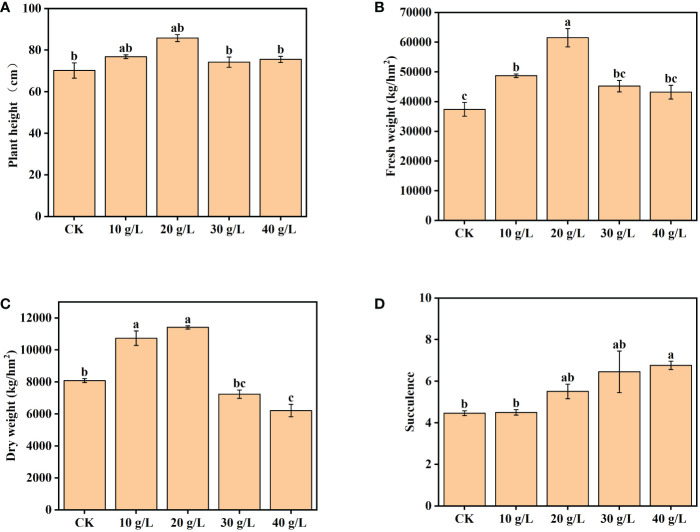
Effects of irrigation water salinity on **(A)** plant height, **(B)** fresh weight, **(C)** dry weight, and **(D)** succulence of *Suaeda salsa*. Bars and error bars indicate the mean ± SE (*n* = 4). Different lowercase letters indicate a significant difference (*p < *0.05).

The growth of *S. salsa* was significantly promoted by low-salinity irrigation water (*p < *0.05). Plant height and aboveground biomass (both FW and DW) initially increased and then decreased with the increase in irrigation water salinity, and the maximum was attained at 20 g/L (**Figure 2**). In the 20 g/L treatment, the plant height, FW, and DW aboveground biomass of *S. salsa* were higher than those of the control by 22.23%, 64.53%, and 41.11%, respectively. In the treatment with irrigation water of 30 g/L or 40 g/L salinity, the plant height, and FW and DW aboveground biomass decreased compared with lower-salinity irrigation water, but were still higher than those of the control. The degree of succulence of *S. salsa* increased significantly with the increase in irrigation water salinity (*p < *0.05), as evident in the succulence of the leaves.

### 3.2 Forage nutritive value of *S. salsa*

#### 3.2.1 Composition

**Figure 3:**
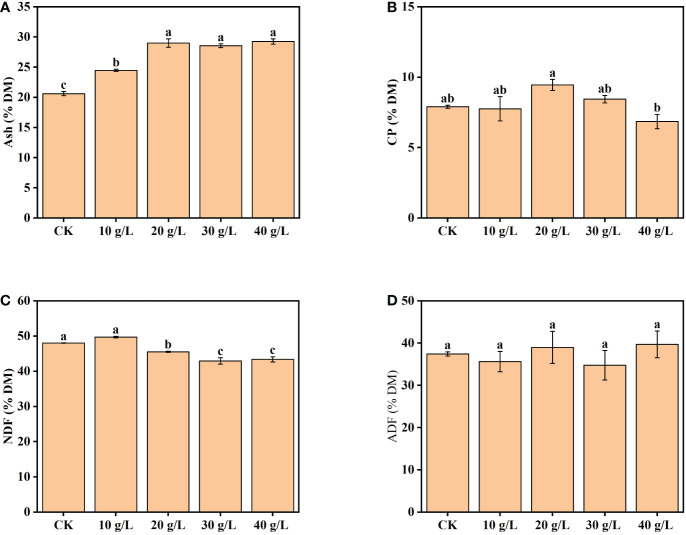
Effects of irrigation water salinity on the contents of **(A)** ash, **(B)** crude protein (CP), **(C)** neutral detergent fiber (NDF), and **(D)** acid detergent fiber (ADF) of *Suaeda salsa*. Bars and error bars are the mean ± SE (*n* = 4). Different lowercase letters indicate a significant difference (*p < *0.05).

The content of ash increased from 20.6% dry matter (DM) to 29.2% DM with increased salinity of the irrigation water from freshwater (CK) to high salinity (40 g/L) (**Figure 3A**). The CP content increased initially, then decreased, with increase in irrigation water salinity (**Figure 3B**). The maximum CP content was observed under moderate salinity (20 g/L). The content of NDF was 42.93%–50.00% DW and decreased significantly under irrigation with water of a salinity of 20 g/L and higher (**Figure 3C**). The ADF content ranged from 35.62% DW to 39.70% DW and was not significantly affected by the salinity of the irrigation water (**Figure 3D**).

#### 3.2.2 Mineral and heavy metal composition of *S. salsa*

Halophytes, which grow naturally in saline environments, generally have higher mineral contents than other plants. Irrigation with brine had a significant effect on the ion content of *S. salsa* (*p < *0.05) (**Table 3**).

**Table 3:** Mineral and heavy metal contents of *S. salsa* under irrigation with water of different salinities.

Ion	CK	10 g/L	20 g/L	30 g/L	40 g/L
K^+^	10.39 ± 0.55c	12.03 ± 0.42bc	13.7 ± 0.98b	16.8 ± 0.70a	16.49 ± 0.49a
Ca^2+^	7.94 ± 0.25b	7.40 ± 0.18b	7.16 ± 0.36b	9.66 ± 0.61a	9.74 ± 0.34a
Na^+^	29.32 ± 5.44b	46.96 ± 1.55ab	42.43 ± 0.38ab	59.27 ± 7.18a	54.97 ± 8.96a
Mg^2+^	13.40 ± 0.22b	11.04 ± 0.42b	12.63 ± 0.82b	15.86 ± 0.52a	17.61 ± 0.86a
Cl^–^	77.67 ± 11.74b	95.88 ± 2.17ab	92.33 ± 4.86ab	119.63 ± 17.20a	126.82 ± 3.65a
S^+^	4.97 ± 0.10c	4.99 ± 0.19c	5.70 ± 0.33bc	6.42 ± 0.46ab	7.20 ± 0.14a
P	734.85 ± 59.56c	817.31 ± 132.10c	1227.93 ± 71.04b	1223.71 ± 37.88b	1689.25 ± 105.43a
Fe^2+^	131.08 ± 10.24a	129.09 ± 3.67a	145.17 ± 1.74a	150.75 ± 26.06a	134.75 ± 7.65a
Cu^2+^	23.07 ± 9.75a	36.91 ± 19.82a	82.75 ± 46.51a	135.39 ± 88.69a	145.55 ± 58.02a
Zn^2+^	35.23 ± 1.28d	58.60 ± 3.76cd	65.62 ± 11.41bc	86.07 ± 7.47b	111.11 ± 1.89a
Mo	0.96 ± 0.02b	1.01 ± 0.02b	1.02 ± 0.03b	1.14 ± 0.10ab	1.48 ± 0.18a
Ni^2+^	2.22 ± 0.27a	0.20 ± 0.20a	0.33 ± 0.33a	0.74 ± 0.74a	1.17 ± 0.70a
Cr^2-^	1.01 ± 0.27a	1.06 ± 0.25a	1.38 ± 0.36a	1.33 ± 0.50a	2.42 ± 0.65a
Cd^2+^	0.18 ± 0.04b	0.24 ± 0.02ab	0.32 ± 0.04ab	0.31 ± 0.04ab	0.35 ± 0.04a
As^3+^	0.18 ± 0.04ab	0.06 ± 0.01b	0.2 ± 0.09ab	0.13 ± 0.00b	0.35 ± 0.04a
Pb^2+^	1.09 ± 0.30a	1.06 ± 0.62a	2.75 ± 1.83a	4.63 ± 3.41a	4.40 ± 2.45a
Hg^2+^	0.02 ± 0.00a	0.02 ± 0.00a	0.02 ± 0.00a	0.03 ± 0.00a	0.02 ± 0.00a

The data are the mean ± SE. Different lowercase letters within a row indicate a significant difference (*p* < 0.05). The unit for K^+^, Ca^2+^, Na^+^, Mg^2+^, and Cl^–^ is g/kg DW. The unit for the other ions is mg/kg DM. CK, freshwater; DM, dry matter; DW, dry weight.

The concentration of macro elements, including Na, K, Ca, Mg, P, and Cl, increased with the increase in irrigation water salinity. The cation and anion with the highest concentrations were Na^+^ (2.93%–5.50% DW) and Cl^–^ (7.77%–11.60% DW), and the highest concentrations were, respectively, 2.02 times and 1.63 times higher than in the control.

Salinity promoted the absorption and accumulation of Cu and Zn. Compared with the control, the Cu and Zn contents increased by 5.9 times and 3.9 times, respectively. The content of Fe (129.09– 150.75 mg/kg DW) did not respond to an increase in irrigation water salinity.

The content of heavy metals increased with the increase in irrigation water salinity. However, with reference to the national standard “Hygienical Standard for Feeds” (GB13078-2017), the concentrations were below the maximum permitted limit and did not pose a risk to human health.

#### 3.2.3 Secondary metabolites

**Figure 4:**
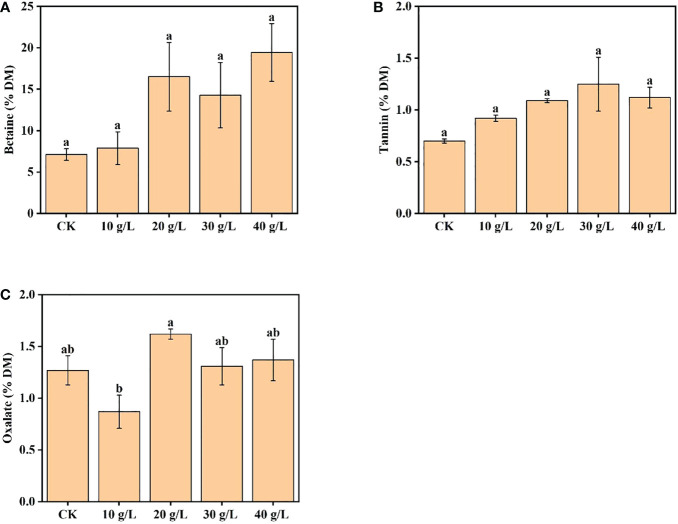
Effects of irrigation water salinity on contents of **(A)** betaine, **(B)** tannin, and **(C)** oxalate of *Suaeda salsa*. Bars and errors bars indicate the mean ± SE (*n *= 4). Different lowercase letters indicate a significant difference (*p < *0.05).

The betaine content increased from 7.13% DW to 19.44% DW with increased salinity of the irrigation water. Compared with the control, the betaine content of plants irrigated with 40 g/L saline increased 2.73 times (**Figure 4A**). The content of tannin first increased and then decreased with increase in irrigation water salinity; the maximum tannin content was observed under moderate salinity (30 g/L) (**Figure 4B**). Under irrigation with water of 40 g/L salinity, the tannin content decreased, but the content was still higher than that of the control. The oxalate content ranged from 0.87% DW to 1.62% DW, which would not reduce feed intake or pose a risk to human health (**Figure 4C**).

### 3.3 Salt removal capacity of *S. salsa* at different developmental stages

The effect of saline water irrigation on the ash content of *S. salsa* was consistent at all growth stages (**Table 4**). The ash content increased significantly with increase in irrigation water salinity from the control (freshwater) to 40 g/L irrigation water concentration. The change in ash content during the entire growth period was consistent under the different treatments. The ash content decreased from the bud stage to the fruit stage.

**Table 4:** Ash content and concentration of *S. salsa* under irrigation with water of different salinities.

Treatment	Bud stage		Flower bud stage		Fruit stage	
	Ash content (% DW)	Ash accumulation (kg/hm^2^)	Ash content (% DW)	Ash accumulation (kg/hm^2^)	Ash content (% DW)	Ash accumulation (kg/hm^2^)
CK	25.25 ± 0.88c	1181.21 ± 49.70b	20.61 ± 0.35c	1666.62 ± 25.51d	17.67 ± 0.09b	3539.76 ± 155.42b
10 g/L	33.95 ± 0.45b	1484.12 ± 37.79a	24.47 ± 0.20b	2627.76 ± 111.75b	21.90 ± 0.44a	5316.86 ± 389.90a
20 g/L	37.30 ± 0.22a	1394.55 ± 92.54a	28.99 ± 0.70a	3307.99 ± 27.57a	22.47 ± 0.69a	5671.03 ± 269.02a
30 g/L	36.93 ± 1.49ab	898.75 ± 24.54c	28.54 ± 0.31a	2065.14 ± 74.08c	21.87 ± 0.32a	5657.56 ± 299.24a
40 g/L	36.35 ± 0.3ab	784.18 ± 60.29c	29.24 ± 0.44a	1816.70 ± 113.45cd	22.81 ± 0.50a	5607.76 ± 458.41a

The data are the mean ± SE. Different lowercase letters within a column indicate a significant difference (*p* < 0.05). CK, freshwater; DM, dry matter; DW, dry weight.

From the bud stage to the fruit stage, although the ash content decreased, the total accumulation of ash increased because of the gradual accumulation of biomass. The ash accumulation increased first and then decreased at all growth stages, which was similar to the trend observed for accumulation in biomass.

**Figure 5:**
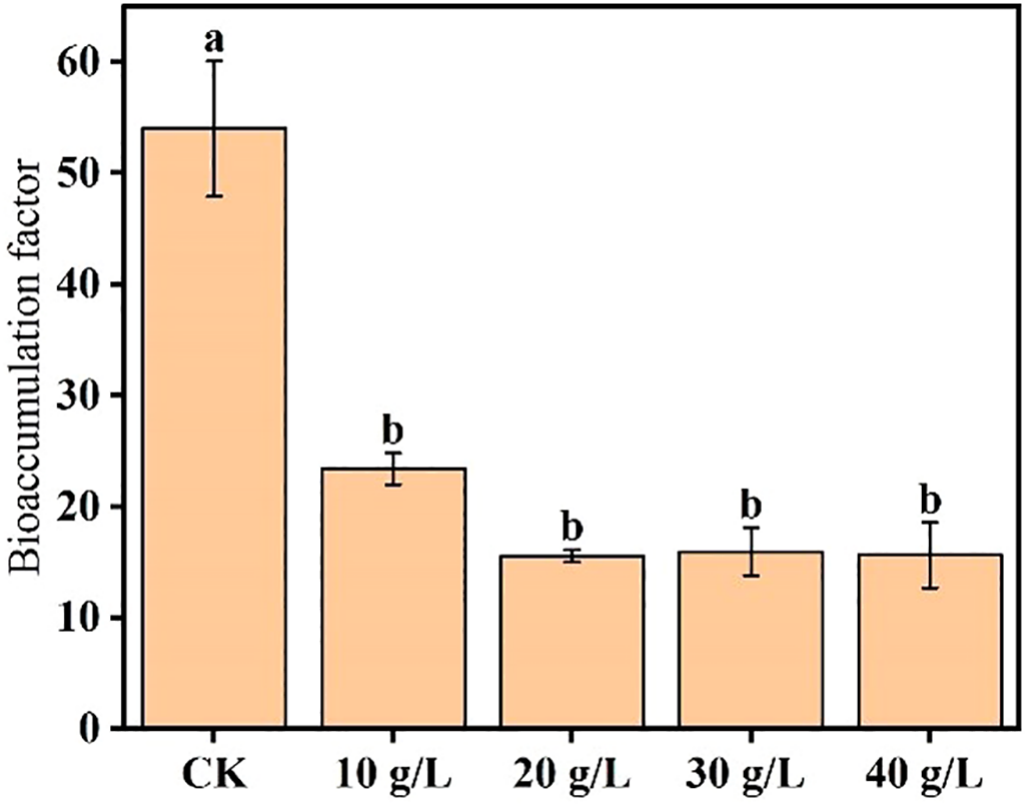
Effects of irrigation water salinity on the salt bioaccumulation factor of *Suaeda salsa*. Different lowercase letters indicate a significant difference (*p* < 0.05).

Ash accumulation is mainly affected by biomass, and ash content is significantly affected by environmental salinity. To compare the plant’s relative salt removal capacity while subjected to the different treatments, its BCF was calculated to assess its ability to absorb salt from the soil. The mean BCF calculated in the different treatments at the flower bud stage is shown in **Figure 5**. The BCF significantly decreased with increase in salinity of the irrigation water (*p* < 0.05) and stabilized at the highest salinities (20 g/L and higher).

## 4 Discussion

### 4.1 Intermediate salinity can improve the growth of *S. salsa*

The aboveground biomass is an important factor in the selection of a halophyte as a forage plant, as it affects the salt removal capacity of the plant. From the perspective of forage, it is meaningless to discuss the nutritive value if substantial biomass, generally measured by DW, is not formed. Based on the present data for growth indicators of *S. salsa*, the forage yield first increased and then decreased with increase in irrigation water salinity, and the peak yield was observed at a salinity of 20 g/L. These results conform to a typical “curvilinear” growth response to salt stress in halophytes, with peak growth observed under moderate salinity (Ma et al., 2019). This is because halophytes have evolved mechanisms by which high concentrations of salt in the environment can be used to their benefit (Guo et al., 2006). Appropriate salt concentrations can promote the vegetative growth of halophytes and are conducive to the completion of their life cycle (Yuan et al., 2019). Among the five treatments in this study, the 20 g/L irrigation concentration may be the most suitable treatment for the growth of *S. salsa*. Morphologically, halophytes have evolved several specific structures. Leaf succulence, which enables accumulation of excessive salt concentrations and conserves water, is a typically visible characteristic of *S. salsa* under high levels of salinity (Zhao et al., 2020). Succulence in itself is not of importance to ruminants (Norman et al., 2013), but can represent the growth state of plants under complex abiotic stress. Ma et al. (2019) argue that succulence could be caused by changes in cell size as a consequence of improved osmotic adjustment. However, in a saline environment, the photosystem of *S. salsa* can be maintained at its normal state and the concentrations of some ions that accumulate in the leaves can promote photosynthetic capacity, which may be one reason for the increase in its aboveground dry weight under salinity (Li et al., 2022).

The annual biomass production of halophytes ranges from 400 to 40,000 kg/hm^2^ in a saline environment (Norman et al., 2013) because the degree of salt tolerance of forage species varies widely. Chenopods are generally more salt tolerant than halophytic grasses and legumes, and as such are widely recognized to show potential for utilization of saline land and water (Masters et al., 2010; Norman et al., 2013). In the present study, the growth of *S. salsa* was promoted by irrigation water of moderate salinity. The annual biomass production ranged from 6,213.07 to 11,410.79 kg/hm^2^; thus, *S. salsa* is potentially suitable as a forage resource. Notably, this yield was achieved under deficit irrigation (irrigation quota: 1,760 m^3^/hm^2^), and would be expected to increase significantly under appropriate agronomic management, such as the supply of appropriate nitrogen and/or adequate water (X. Wang et al., 2021). These results show that *S. salsa* can produce substantial biomass for use as a potential source of forage. Thus, *S. salsa* can fill the annual feed shortage, and allow farmers to earn income from saline wasteland.

### 4.2 Moderate salinity can improve the forage nutritive value of *S. salsa*

In addition to yield, forage nutritive value, mainly referring to the CP, fiber, and ash content, is an important aspect of forage (Al-Dakheel et al., 2015). For the same species in the same growth period, it is generally believed that the treatment with the highest CP content and lowest fiber content has the highest forage nutritive value (Jungers et al., 2020).

In the present study, the CP content of *S. salsa* first increased to 9.45% DW and then decreased to 6.85% DW with increase in the irrigation water salinity. Abundant salt-responsive proteins, which contribute to diverse functions such as photosynthesis, osmotic and ionic homeostasis, signal transduction, and reactive oxygen species-scavenging systems, have been identified (Kumari et al., 2015). The increase in CP content is an essential feature of salt tolerance (Koyro et al., 2013). Hence, there is a general tendency for the abundance and activity of transporter proteins to increase under hyperosmotic salinity initially; these proteins then decrease as the external salt concentration continues to increase. This decrease in CP content is an indicator of severe stress and leads to the eventual death of the plant (Koyro et al., 2013), which is consistent with the present results. Decreased CP content in a saline environment may be due to the reduction in nutrient uptake from saline soils, which leads to reduced synthesis or enhanced degradation of proteins, low amino acid availability, and the denaturation of enzymes associated with protein synthesis (Hedayati-Firoozabadi et al., 2020). Previous studies have reported the CP content in a range of halophytic plants, ranging from 3.38% DM to 15.1% DM (El Shaer, 2010; Pirasteh-Anosheh et al., 2021). In this study, the CP content of *S. salsa* is higher than that of some halophytic forage considered to provide high-protein forage., There is no unified standard for the CP content of forage, as requirements vary among animals and growth stages, and the protein requirement of ruminants for maintenance, growth, and reproduction is low (Hessini et al., 2020). A plant with a CP content > 7% is considered to be highly suitable as a forage plant, and a plant with a CP content of 5%–7% is considered suitable (Pirasteh-Anosheh et al., 2021). Hence, in the present study, *S. salsa* can be regarded as a high-quality forage or protein supplement for ruminants in arid–saline rangelands. In addition, low CP contents may be improved through agronomic means (Norman et al., 2013).

Halophytes are characterized by having a high content of indigestible fiber (Norman et al., 2013), which generally reduces the forage nutritive value by reducing livestock intake and the digestibility of most nutrients (Harper and McNeill, 2015; Waldron et al., 2020). In the current study, the NDF decreased significantly under irrigation with water of salinity 20 g/L and above, whereas the ADF content was not significantly affected by irrigation water salinity. According to existing research, interactions between the salinity of the environment and the fiber content of grasses are inconsistent. Pasternak et al. (1993) observed no consistent relationship between the fiber content of five halophytic grasses and soil salinity. Robinson et al. (2004) reported that the NDF content of *Cynodon dactylon* increased with salinity, whereas that of *Thinopyrum ponticum* decreased. Hence, the relationship between fiber content and salt stress requires further study. Based on the results of Pirasteh-Anosheh et al. (2021), an ADF content of > 45% is not suitable and 35% < ADF < 45% is suitable, whereas an NDF content > 50% is not suitable and 40% < NDF < 50% is suitable. Accordingly, both the NDF (42.93%–50.00% DM) and ADF (34.76%–39.70% DM) contents of *S. salsa* in the present study were suitable. Trace elements, such as Zn, Cu, and Fe, are involved in most biochemical metabolic processes of the body, as they are the main components of cellular enzymes and transcription factors in animals, and should be given priority when evaluating forage nutritive value (Fang et al., 2013). Xinjiang comprises a vast land area, and mineral elements are widely but unevenly distributed (Li et al., 2017). Herbivorous livestock in Xinjiang are generally deficient in elements such as Cu and Zn, which is a major limiting factor for the development of animal husbandry in the province. Livestock are not commonly fed mineral supplements, either individually or in blocks by way of licks, because of the high cost of such supplements (Gowda et al., 2004). Forage halophytes may be a source of certain mineral elements to meet livestock demands. In the present study, Cu and Zn contents were higher in *S. salsa* plants than in other forage grasses, and were significantly increased after irrigation with saline water. As a result, *S. salsa* forage is an ideal source of mineral supplements for livestock.

### 4.3 Potential feeding risks of *S. salsa* irrigated with saline water

A high ash content can decrease forage nutritive value by affecting palatability, consumption, and nutrient utilization (El Shaer, 2010). A high ash content is a typical characteristic of halophytes. Hence, the ash content can be the main limiting factor for the utilization of halophytes as forage. In this study, the ash content increased with increased salinity of the irrigation water. Similarly, Hessini et al. (2020) reported that species of plants in the Chenopodiaceae family have a much higher ash content in their DW than salt-tolerant grass species, and further suggested that a high ash content is a major factor restricting the forage nutritive value of *S. salsa*.

Ash content reflects a high mineral content, and mineral composition is associated with selective absorption by the plant and the soil properties (Zhang et al., 2012). In the present study, the Na_11_ and Cl contents were highest in *S. salsa*. In general, an increase in Na accumulation corresponds with decreases in K, Mg, and Ca contents (Waldron et al., 2020). However, *S. salsa* is unique in the genus *Suaeda* with respect to K responses; this species has developed high selectivity for K over Na by the roots, and thus can absorb and accumulate large amounts of K in plant tissues to meet the nutrient requirements of the plant (Mori et al., 2011). The K/Na, Mg/Na, and Ca/Na ratios decreased with increased salinity of the irrigation water, which suggests that the increase in K, Mg, and Ca contents of *S. salsa* might be due to an increase in total mineral content.

A high salt concentration (Na and Cl) can reduce the forage nutritive value of *S. salsa* by contributing to a reduction in feed and DM intake and compromising animal health (Hessini et al., 2020; Waldron et al., 2020). In the present study, the salt content accumulated to a level that exceeds the tolerance threshold for livestock (up to 100 g/kg) (Hessini et al., 2020). Thus, *S. salsa*, similar to other halophytic forage, is not suitable for direct use as a staple food source, and should be used in the form of silage or/and mixed with other forage (El Shaer, 2010; Norman et al., 2013). In addition, offering fresh drinking water to animals would reduce the stress of salt intake and enhance halophyte consumption and nutrient utilization (El Shaer, 2010; Runa et al., 2019). This increase may be caused by increased palatability of feed and enhanced digestion owing to the stimulation of microbial activities (Runa et al., 2019). Moreover, salt in the diet can have both positive and negative impacts, depending on the concentration (Hessini et al., 2020). Thus, when administered at an appropriate concentration, *S. salsa* could be used as a natural salt supplement.

Growing in a saline environment, halophytes use organic acids to balance the excessive absorption of anions, and the most abundant of these is oxalate (Masters et al., 2001). Thus, the oxalate content of halophytes, especially that of the chenopodiaceous salt-tolerant shrub, was close to the toxicity threshold (Li et al., 2017). High oxalate concentrations can reduce the availability of mineral nutrients for rumen microflora and the host animal by forming complexes with Ca, Mg, and possibly other minerals, which may lead to calcium deficiency, kidney damage, and, finally, death (El Shaer, 2010; Norman et al., 2013; Hessini et al., 2020). In addition, a more common observation is that a high oxalate concentration can lead to a decrease in feed intake. In this study, the oxalate concentration ranged from 0.87% to 1.62%, and was within the safety threshold (2%).

Tannins, as phenolic plant secondary compounds, represent a “double-edged sword” for forage nutritive value. Condensed tannins (CTs) protect proteins from excessive degradation, but also inhibit the absorption of protected proteins (Waghorn, 2008), depending on their concentration in the feed material. El Shaer (2010) reported that low concentrations of tannins (i.e., 2%–4%) in the diet increase the absorption of essential amino acids in ruminants, whereas higher tannin concentrations (i.e., 4%–10%) decrease forage intake. Zhang et al. (2009) reported that a plant with a tannin concentration of > 3% may limit feed intake and reduce digestibility by ruminants. However, the appropriate concentration and toxicity threshold are dependent on the species and physiological status of ruminants (Waghorn, 2008). For example, the beneficial effects of CTs are observed in the range 2.2%–3.8% DM, and the action of CTs in grazing lactating ewes has been reported to affec milk secretion only in mid and late lactation (Min et al., 2003). In the present study, the tannin content increased with the increase in salinity of the irrigation water, but did not exceed the current safe toxicity threshold.

Therefore, as forage, *S. salsa* poses certain potential risks owing to its excessive ash content, salt accumulation, mineral imbalance, and the presence of anti-nutritional factors. However, these risks should not discourage the use of *S. salsa* in agricultural contexts, as these potential risks might be diminished through the application of specific physical treatments (e.g. chopping, soaking and sun-drying), chemical treatments (e.g. PEG) and biological treatments (e.g. ensiling) (El Shaer, 2010). In conclusion, halophytes such as *S. salsa* have broad prospects as livestock and agronomists should aim to maximize the forage nutritive value.

### 4.4 Ecological significance of planting *S. salsa*

Halophytes occur naturally in high-salinity areas and can absorb salt from the rhizosphere. Salt accumulates to high concentrations in the tissues of halophyte plants, and, thus, halophytes can be used for the remediation of salt-affected land (Congjuan et al., 2018; Shaygan et al., 2018). The accumulation of salt, which is affected by plant aboveground biomass and ion concentration, is an important factor with which to measure the ability of halophytes to improve saline soil (Wang et al., 2022). Based on its salt removal capacity (measured by salt accumulation) and BCF, *S. salsa* is an effective salt absorber in saline soils. *Suaeda salsa* might significantly reduce the soil salinity if harvested at the end of the growing season (Zhao, 1991). In the present study, the aboveground biomass was an important factor affecting salt accumulation. Wu et al. (2012) reported that *S. salsa* grows rapidly in moderate-salinity soil and can survive extreme salinities, which is consistent with our findings in the present study. Salt accumulation ranged from,1666.62 to 3,307.99 kg/hm^2^ at the flower bud stage and from 3,539.76 to 5,771.03 kg/hm^2^ at the fruit stage, values that are similar to those reported previously. Liang and Shi (2021) reported that *S. salsa* can remove 3.0–3.8 t/ha of Na, and that its salt extraction potential ranges from 3.75–3.91 t/ha/year. Therefore, these studies, including the present, suggest that *S. salsa* is an ideal species for the artificial revegetation of high-salinity land.

In the present study, to study the growth and forage nutritive value of *S. salsa* under extreme irrigation conditions, we used irrigation water with a salinity as high as 40 g/L. *S. salsa* did not improve the salinity of soil when harvested at the initial flower bud stage. In practical agricultural production, brackish water is often used for irrigation. However, according to the salt balance formula (Zhao et al., 2013), the salt removed from the soil was greater than that introduced by irrigation under the control treatment (freshwater). Therefore, when irrigated with local saline water, *S. salsa* has a huge capacity for improving saline soil, in addition to providing forage for livestock.

Water of high salinity is mainly used for revegetation and ecological restoration in extremely saline–alkali land. In this context, *S. salsa* can be harvested at the fruit stage, once greater biomass has accumulated, to remove greater quantities of salt from the soil. Even if it is not able to reduce the salt content of the soil, it will provide stable vegetation cover. Vegetation cover on the soil surface is a major factor that affects soil moisture content and temperature. *S. salsa* can survive in highly saline environments and replaces soil surface evaporation with its own evapotranspiration. This “mulching effect” of halophytes can inhibit the return of soil salt and transform the environment so that it is more directly conducive to plant growth (Shaygan et al., 2018), and, thereby, improve the ability of the soil to resist saline–alkali damage. In addition, the interspersed roots of salt-tolerant plants improve the physical properties of saline–alkali soil (X. Wang et al., 2021). X. Wang et al. (2021) reported that the absolute water content, total porosity, and capillary porosity were increased after the planting of halophytes. *S. salsa*, having a taproot system, has the potential to act as a tillage tool (Chaudhary et al., 2018). Such biological drilling can stabilize the soil structure by reducing the soil bulk density, thereby improving salt ion leaching from saline–alkali soil by increasing the soil porosity and hydraulic conductivity, and reducing the bulk density (Rahman et al., 2021). In addition, halophytes exert a significant influence on the soil’s physical and chemical properties *via* root exudation and litter decay (Mao et al., 2014; Chaudhary et al., 2018; Ping et al., 2018). Mao et al. (2014) reported that the presence of *S. salsa* altered the distribution of soil aggregate size in tidal salt marshes, and increased the concentrations of soil organic C and total N in the salt tidal marshes of the Liaohe Delta. Li et al. (2019) observed that the planting of *S. salsa* in the Tianjin Estuary area, China, increased the soil organic matter content by 43.4% and the total soil N content by 17.8%, and greatly increased the soil microbial amount. In addition, after halophyte planting, the soil microbial species, abundance, microbial activity, and evenness were greatly improved (X. Wang et al., 2021), and the roots of halophytes induced the synthesis of these enzymes (Rathore et al., 2017).

## 5 Conclusion

The present results show that *S. salsa* can tolerate irrigation water with a salinity of 40 g/L. Therefore, based on its salt tolerance, *S. salsa* has strong potential for the development of saline agriculture strategies and to improve the natural ecosystem. Under irrigation with a low concentration of saline water, *S. salsa* can provide high-quality forage and reduce the salt concentration of the soil. The plant’s salt removal capacity, as measured by salt accumulation in aboveground tissues, is affected by biomass production and ash content. Therefore, harvesting it at the end of its growth stage, when its biomass is maximal, enables *S. salsa* to reduce the soil salt content to the greatest extent. Under the present experimental conditions, the highest quality forage can be obtained by irrigation with water of 20 g/L salinity. Specifically, at this salinity, the maximum biomass and CP content were attained and minimum fiber content was observed. In addition, trace element contents were improved by irrigation with saline water. However, it cannot be asserted that 20 g/L is the optimal irrigation water salinity for the growth and forage nutritive value of *S. salsa*, because the soil environment is complex and diverse, and plant growth is affected by various factors. Nevertheless, it is certain that this plant’s biomass will increase in a suitable saline environment, and salinity can increase its forage nutritive value by increasing the CP content and mineral nutrition, and decreasing the fiber concentration. In addition, high-salinity water can be used for revegetation in a saline wasteland. Even if it is not able to reduce the salt content of the soil, planting with *S. salsa* provides a stable vegetation cover that can transform the environment to improve its physicochemical properties for plant growth. However, the salt tolerance threshold of *S. salsa* and its influence on revegetation need further investigation.

## Data availability statement

The raw data supporting the conclusions of this article will be made available by the authors, without undue reservation.

## Author contributions

NW: conceptualization, investigation, and writing of original draft. ZZ: conceptualization, supervision, and writing (i.e., reviewing and editing). XZ: investigation. SL: investigation and writing of original draft. KZ: investigation and supervision. MH: resources. All authors contributed to the article and approved the submitted version.
